# Role of IL-6 in Exercise Training- and Cold-Induced UCP1 Expression in Subcutaneous White Adipose Tissue

**DOI:** 10.1371/journal.pone.0084910

**Published:** 2014-01-08

**Authors:** Jakob G. Knudsen, Maria Murholm, Andrew L. Carey, Rasmus S. Biensø, Astrid L. Basse, Tamara L. Allen, Juan Hidalgo, Bronwyn A. Kingwell, Mark A. Febbraio, Jacob B. Hansen, Henriette Pilegaard

**Affiliations:** 1 Centre of Inflammation and Metabolism, Department of Biology, University of Copenhagen, Copenhagen, Denmark; 2 Department of Biology, University of Copenhagen, Copenhagen, Denmark; 3 Department of Biomedical Sciences, University of Copenhagen, Copenhagen, Denmark; 4 Metabolic and Vascular Physiology Laboratory, Baker IDI Heart and Diabetes Institute, Melbourne, Victoria, Australia; 5 Cellular and Molecular Metabolism Laboratory, Baker IDI Heart and Diabetes Institute, Melbourne, Victoria, Australia; 6 Department of Neuroscience, Universidad Autonoma de Barcelona, Barcelona, Spain; INSERM/UMR 1048, France

## Abstract

Expression of brown adipose tissue (BAT) associated proteins like uncoupling protein 1 (UCP1) in inguinal WAT (iWAT) has been suggested to alter iWAT metabolism. The aim of this study was to investigate the role of interleukin-6 (IL-6) in exercise training and cold exposure-induced iWAT UCP1 expression. The effect of daily intraperitoneal injections of IL-6 (3 ng/g) in C57BL/6 mice for 7 days on iWAT UCP1 expression was examined. In addition, the expression of UCP1 in iWAT was determined in response to 3 days of cold exposure (4°C) and 5 weeks of exercise training in wild type (WT) and whole body IL-6 knockout (KO) mice. Repeated injections of IL-6 in C57BL/6 mice increased UCP1 mRNA but not UCP1 protein content in iWAT. Cold exposure increased iWAT UCP1 mRNA content similarly in IL-6 KO and WT mice, while exercise training increased iWAT UCP1 mRNA in WT mice but not in IL-6 KO mice. Additionally, a cold exposure-induced increase in iWAT UCP1 protein content was blunted in IL-6 KO mice, while UCP1 protein content in iWAT was lower in both untrained and exercise trained IL-6 KO mice than in WT mice. In conclusion, repeated daily increases in plasma IL-6 can increase iWAT UCP1 mRNA content and IL-6 is required for an exercise training-induced increase in iWAT UCP1 mRNA content. In addition IL-6 is required for a full induction of UCP1 protein expression in response to cold exposure and influences the UCP1 protein content iWAT of both untrained and exercise trained animals.

## Introduction

Uncoupling protein 1 (UCP1) is primarily expressed in brown adipose tissue (BAT) and is central to the thermogenic function of the tissue [1]. Expression of UCP1 in white adipose tissue (WAT) has been observed in response to treatment with a β3-adrenergic receptor agonist [2] and cold exposure [3].This has led to the suggestion that expression of BAT genes in WAT and concomitant browning of the tissue may alter WAT metabolism [4]. BAT UCP1expression is induced by cold exposure through activation of the sympathetic nervous system [5]. Transcription factors and cofactors such as peroxisome proliferator activated receptor γ (PPARγ) and the PPARγ co-activator-1α (PGC-1α) mediate this activation of UCP1 transcription [6], [7] and previous studies suggest that such regulation of UCP1 expression also occurs in iWAT [2], [3]. Exercise and exercise training have recently been shown to increase UCP1 mRNA and/or protein content in inguinal WAT (iWAT) [8], [9], but not in epididymal WAT [8]. In addition, expression of other BAT related genes such as cell death-inducing DFFA-like effector A (*Cidea*) and elongation of very long chain fatty acids 3 (*Elovl3*) also increases in iWAT with exercise training and/or iWAT browning [9]. Cleaved fibronectin type III domain containing 5 (FNDC5) released from skeletal muscle as the myokine irisin was suggested to increase UCP1 mRNA content in iWAT during exercise [9]. The previous finding that exercise training increases the basal plasma concentration of irisin in mice [9] clearly indicates the potential impact of irisin in exercise training-induced iWAT browning, however other factors may be involved in the regulation of iWAT browning in response to exercise training.

The cytokine interleukin 6 (IL-6) is released from contracting skeletal muscle to the circulation [10] and previous observations suggest that IL-6 elicits metabolic effects in adipose tissue [11], [12]. Additionally, exercise trained IL-6 knockout (KO) has been reported to have increased iWAT PPARγ mRNA relative to exercise trained wild type (WT) mice [13] suggesting increased adipogenesis in the absence of IL-6 [14]. This is supported by previous observations that IL-6 KO mice have higher body weight than WT mice due to increased iWAT mass [13], [15]. Another study has, however, shown no increase in body weight in IL-6 KO mice compared with WT mice [16]. The decreased oxygen consumption and increased respiratory exchange ratio at room temperature observed in IL-6 KO mice relative to WT mice [17], indicating reduced whole body energy expenditure and fat oxidation in IL-6 KO mice, support that mice lacking IL-6 have dysfunctional regulation of metabolism. Interestingly, IL-6 KO mice have also been shown to have lower core body temperature than WT mice when housed at 4°C without a decrease in UCP1 expression in interscapular BAT (iBAT) [17]. This may suggest that cold-induced UCP1 expression and thermogenesis in WAT are lower in IL-6 KO than in WT mice.

Together the above studies indicate that IL-6 may play a role in iWAT metabolism, through the regulation of UCP1 in response to exercise training and cold exposure. Therefore, the aim of the present study was to test the hypothesis that IL-6 is required for the exercise training- and cold-induced increase in UCP1 expression in mouse iWAT.

## Methods

### Ethics statement

The exercise training and injection experiments were carried out in Denmark and approved by the Danish Animal Experimental Inspectorate and complied with the ‘European Convention for the Protection of Vertebrate Animals Used for Experiments and Other Scientific Purposes’ (Council of Europe, no. 123, Strasbourg, France, 1985). The cold exposure experiment was carried out in Australia and approved by The Alfred Medical Research and Education Precinct Animal Ethics Committee.

### Animals

In all experiments male C57BL/6 and/or whole body IL-6 knockout (KO) mice on a C57BL/6 background were used. All animals were housed at 22°C on a 12:12 hour light:dark cycle in the injection study and up to the cold exposure study, or a 11:13 hour light:dark cycle in the exercise training study. The animals had access to standard rodent chow (Altromin no. 1324 Brogaarden, Lynge, Denmark) and water *ad libitum*. Animals were housed in separate cages in the injection study, 2–3 animals per cage in the cold exposure study and in pairs in the training study. The IL-6 KO mice have previously been described [13], [15], [17]–[20].

### Acute IL-6 injection

Eight weeks old C57BL/6 mice received intraperitoneal (IP) injections of either recombinant mouse (rm) IL-6 (3 ng/g) (R&D Systems, Minneapolis, MN, USA) in sterile PBS, or an equal volume of sterile PBS (n = 8). One hour after the injection, the mice were euthanized by cervical dislocation, trunk blood was collected and centrifuged (2600 g, 15 min) to generate plasma and iWAT was removed, frozen in liquid nitrogen and stored at −80°C for later analyses.

### Repeated IL-6 injection

Nine weeks old C57BL/6 mice received IP injections of either rmIL-6 (3 ng/g) (R&D Systems) in sterile PBS, or an equal volume of sterile PBS (n = 12) once per day for 7 days. Twenty-four hours after the last injection, the mice were euthanized by cervical dislocation and iWAT was removed, frozen in liquid nitrogen and stored at −80°C for later analyses.

### Cold exposure study

Eight weeks old C57BL/6 mice and IL-6 KO mice housed at room temperature were randomly divided into two groups, housed at room temperature or at 4°C (n = 12) for 72 h. At the end of the 72 h, the mice were anaesthetized with sodium pentobarbital, blood for serum was collected from the inferior vena cava and iWAT and iBAT was removed, frozen in liquid nitrogen and stored at −80°C for later analyses.

### Exercise training

Four month old C57BL/6 WT and whole body IL-6 KO mice were either exercise trained for 1 h/day, 5 days/week for 5 weeks, or remained untrained (n = 8). Mice ran on a treadmill (Model Exer-4 Treadmill, Columbus Instruments international; Columbus, OH, USA) with 10% incline and the speed initially at 14.9 m/min increasing to 16.7 m/min in the last week. To ensure that the mice stayed on the treadmill, the mice were encouraged by air blown gently from behind with an air gun. Thirty-six hours after the last exercise bout, mice were euthanized by cervical dislocation and iWAT was removed, frozen in liquid nitrogen and stored at −80°C for later analyses. Results obtained from tissue analyses of these mice have previously been published [13], [18], [19].

### Plasma IL-6

Plasma IL-6 concentrations were measured using Meso Scale Discovery (MSD, Rockville, MD, USA) cytokine kit recognizing murine IL-6. Samples were run in duplicate accordingly to the manufacturer's protocol.

### RNA isolation

RNA isolation from the injection and exercise training studies were performed on ∼30 mg of iWAT using a guanidinium-thiocyanate-phenol-chloroform protocol [21] with modifications [22]. RNA isolation on samples from the cold exposure was performed using TRIzol as described by the manufacturer (Life Technologies, Naerum, Denmark).

### Reverse transcription and PCR

Reverse transcription on samples from the injection and exercise training protocols was performed using Superscript II RNase H^−^ system and oligodT (Life Technologies) as previously described [22]. Reverse transcription of samples from the cold exposure experiment was performed using MMVL reverse transcriptase (Life Technologies) and random hexamers (DNA technology, Risskov, Denmark). Real time PCR on samples from the injection and exercise training experiments was performed using The ABI 7900 Sequencing Detection System (Applied Biosystems, Foster City, California, US). Samples were run in triplicates with primers and TaqMan probes using Universal Mastermix or SYBRGreen (Applied Biosystems). The mRNA sequences were obtained from Ensembl (www.ensembl.org) and primers and TaqMan probes were designed using Primer Express (Applied Biosystems), as listed in Table 1. TaqMan probes were 5′-6-carboxyfluorescein (FAM) and 3′-6-carboxy-N,N,N′,N′-tetramethylrhodamine (TAMRA) labeled. The results from a serial dilution of a pooled representative sample were used to construct a standard curve from which the Ct values of the unknown samples were converted to relative amounts. Samples were normalized to the ssDNA concentration determined by OliGreen as previously described [23] or GAPDH mRNA content.

**Table 1:** Primers and probes.

mRNA	Exercise and injection study	Cold exposure
UCP1	FP: 5′ TGTGCG ATGTCCATGTACACCA 3′	FP: 5′ GGCATTCAGAGGCAAATCAGCT 3′
	RP: 5′ ACCCGAGTCGCAGAAAAGAA 3′	RP: 5′ CAATGAACACTGCCACACCTC 3′
	Probe: 5′ CCACAAACCCTTTGAAAAAGGCCGTC 3′	
PGC-1α	FP: 5′ AGCCAAACCAACAACTTTATCTCTTC 3′	FP: 5′ AGCCGTGACCACTGACAACGAG 3′
	RP: 5′ TTAAGGTTCGCTCAATAGTCTTGTTC 3′	RP: 5′ GCTGCATGGTTCTGAGTGCTAAG 3′
	Probe: 5′ GAGTCACCAAATGACCCCAAGGGTTCC 3′	
Elovl3	FP: 5′ AAGGTTGTTGAACTGGGAGA 3′	FP: 5′ TCGTCTATCTGTTGCTCATCG 3′
	RP: 5′ GGACAAAGATGAGTGGACGCTTA 3′	RP: 5′ CTGTTGCCATAAACTTCCACA 3′
Cidea	FP: 5′ AAAGGGACAGAAATGGACAC 3′	FP: 5′ AAAGGGACAGAAATGGACAC 3′
	RP: 5′ TTGAGACAGCCGAGGAAG 3′	RP: 5′ TTGAGACAGCCGAGGAAG 3′
FNDC5	FP: 5′ GGTCTCCTCCGCAGCAAGATA 3′	
	RP: 5′ AAGGTCCTCTCGCATTCTCTACTGT 3′	
	Probe: 5′ TTCTGAGCTGCTCAGAGCAAGCACTGTAAA 3′	

Primers and probes used in real time PCR.

Real time PCR on samples from the cold exposure experiment was performed using a Stratagene Mx3000P system (Stratagene, La Jolla, CA, USA) with MESA FAST qPCR MasterMix (Eurogentec, Seraing, Belgium). Samples were run in duplicates and primers are listed in Table 1. Results were calculated using the ΔΔCt method and samples were normalized to the mRNA content of TATA box binding protein (TBP).

### Protein lysate preparation

Protein isolation was performed on ∼45 mg of iWAT after homogenization (Tissue Lyzer, Qiagen, Hilden, Germany) in 10% glycerol, 20 mM Na-pyrophosphate, 150 mM NaCl, 50 mM hepes, 1% NP-40, 20 mM β-glycerolphosphate, 10 mM NaF, 1 mM EDTA, 1 mM EGTA, 20 µg/ml Aprotinin, 10 µg/ml leupeptin, 2 mM Na_3_VO_4_ and 3 mM benzamidine adjusted to pH 7.5. Lysates were obtained by centrifugation of the homogenate at 16000 g for 20 min. Before protein determination by the bicinchoninic acid assay (Pierce, Thermo Fisher Scientific, Waltham, MA, USA) 2% SDS was added to lysates to prevent lipid interference in the bicinchoninic acid assay. After protein determination, lysates were adjusted with SDS containing sample buffer to a concentration of 1 µg/µl in the exercise training, cold exposure and acute injection studies and 0.5 µg/µl in the repeated injection study and heated to 96°C for 3 min.

### Western blotting

Samples were separated by SDS-PAGE on a 10% acrylamide gel and blotted on to a PVDF membrane (Millipore, Hellerup, Denmark), blocked in 3% fish gel (Invitrogen, Paisley, UK) in Tris buffered saline with 0.05% tween 20 (pH = 7.4) and incubated overnight in primary antibody recognising either UCP1 protein (AB 10983, Abcam, Cambridge, UK), phosphorylated STAT3 protein (#9145,Cell Signaling, Danvers, MA, USA), STAT3 protein (#9139, Cell Signaling) or β-actin protein (#4967, Cell Signalling). The following day, membranes were incubated in horseradish peroxidase-conjugated polyclonal anti-rabbit secondary antibodies (Dako, Glostrup, Denmark) in 3% fish gel. Bands at ∼33 kDa (UCP1), ∼79 kDa (STAT3) and ∼45 kDa (β-actin) were visualized using luminescence on a Carestream IS 4000IM and quantified using Carestream Health Molecular Imaging software (Fisher Scientific, ThermoFisher Scientific, Waltham, MA, USA).

### Statistics

All data are reported as mean ± standard error (SE). Two-way ANOVA was used to test the effect of cold exposure and genotype as well as exercise training and genotype on iWAT mRNA and protein content. A Student Newman-Keuls post-hoc test was used to locate differences. If normal distribution or equal variance failed, a Mann-Whitney U test was performed. A Students t-test was used to test the effect of a single IL-6 injection on STAT3 phosphorylation and the effects of repeated injections of IL-6 on iWAT mRNA and protein content. A value of p<0.05 was considered significant.

## Results

### Plasma

*IL-6:* Plasma concentration of IL-6 was 13.65±1.91 pg/ml in PBS and 59.79±7.41 pg/ml in IL-6 injected animals 1 h after injection.

Serum concentration of IL-6 was unchanged in WT mice after 3 days at 4°C compared with 22°C with concentrations of 8.93±0.42 pg/ml and 9.62±0.59 pg/ml, respectively.

### Inguinal WAT

#### mRNA

**Figure 1:**
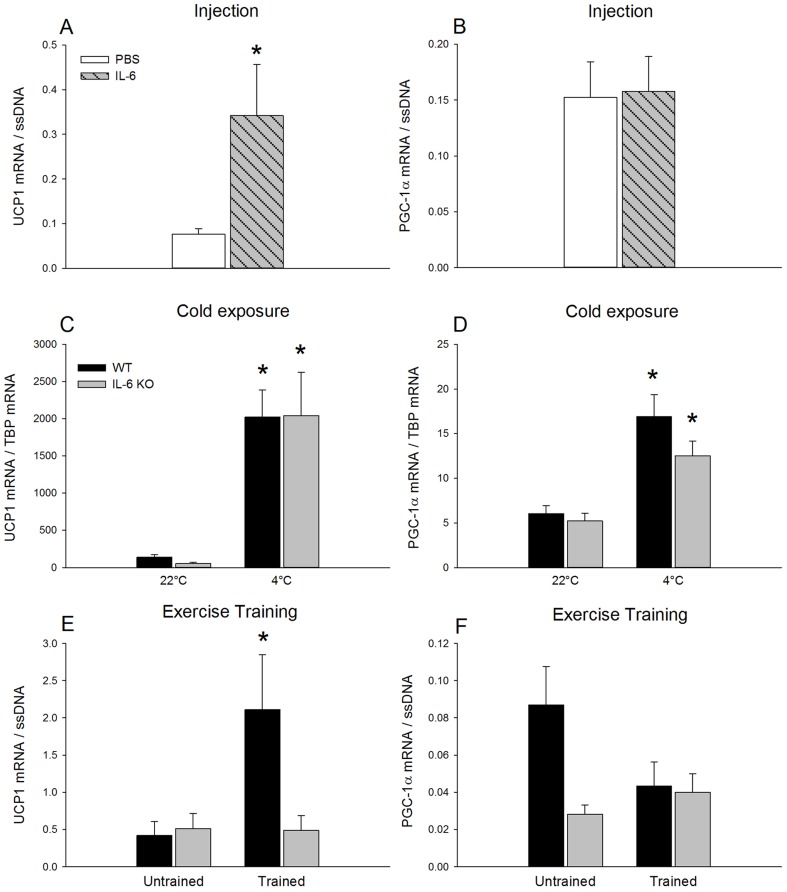
UCP1 and PGC-1α mRNA content in iWAT. UCP1 and PGC-1α mRNA in iWAT after 7 days of daily injection of IL-6 or PBS in C57BL/6 mice (A and B), in response to 3 days of cold exposure in WT and IL-6 KO mice (C and D) and in untrained and trained WT and IL-6 KO mice (E and F). The target mRNA is normalized to single stranded (ss) DNA or TBP mRNA content. Values are mean ± SE, (n = 6–12). *: significantly different from PBS or 22°C (p<0.05).

*UCP1 and PGC-1α:* UCP1 mRNA content in iWAT was ∼5-fold higher (p<0.05) after repeated injections of IL-6 than after PBS injections (Fig. 1A). PGC-1α mRNA (Fig. 1B) did not change with repeated IL-6 injections compared with PBS.

Cold exposure increased (p<0.05) iWAT UCP1 mRNA content ∼9- and ∼19-fold (Fig. 1C) and iWAT PGC-1α mRNA content ∼2.5- and ∼2-fold (Fig. 1D) in WT and whole body IL-6 KO mice, respectively.

The UCP1 mRNA content was ∼5-fold higher (p<0.05) in trained than untrained WT mice, but with no difference between untrained and trained IL-6 KO mice (Fig. 1E). The PGC-1α mRNA content in iWAT was unaffected by exercise training in both WT and IL-6 KO mice (Fig. 1F)

**Figure 2:**
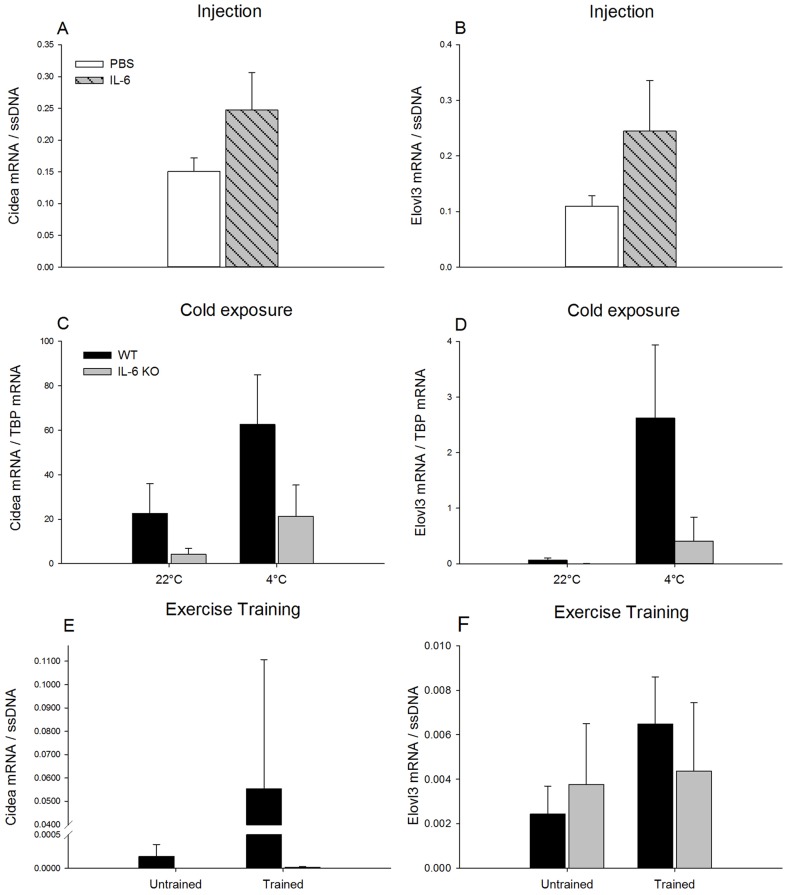
Cidea and Elovl3 mRNA content in iWAT. iWAT Cidea and Elovl3 mRNA content after 7 days of daily injection of IL-6 or PBS in C57BL/6 mice (A and B), in response to 3 days of cold exposure in WT and IL-6 KO mice (C and D) and in untrained and trained WT and IL-6 KO mice (E and F). The target mRNA is normalized to single stranded (ss) DNA or TBP mRNA content. Values are mean ± SE, (n = 6–12).

*Elovl3 and Cidea:* Cidea and Elovl3 mRNA content in iWAT did not change in response to repeated injections of IL-6 compared with PBS (Fig. 2A and B).

Elovl3 and Cidea mRNA content in iWAT was unchanged in response to cold exposure. However it must be noted that the number of animals expressing these two mRNAs varied from 2–11 in each group (Fig. 2C and D). Samples with Ct ≥40 were included as 0 in the calculations.

Elovl3 and Cidea mRNA content in iWAT was unchanged with exercise training, but it should be noted that only 2–4 animals expressed Cidea mRNA and 6–8 expressed Elovl3 mRNA in iWAT in each group, (Fig. 2E and F). Samples with Ct ≥40 were included as 0 in the calculations.

#### Protein

**Figure 3:**
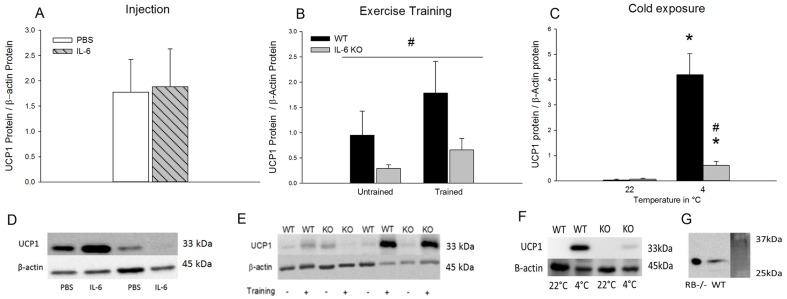
UCP1 protein Content in iWAT. iWAT UCP1 protein content after 7 days of daily injection of IL-6 or PBS in C57BL/6 mice (A), in untrained and trained WT and IL-6 KO mice (B), and in response to 3 days of cold exposure in WT and IL-6 KO mice (C). Representative blots from 7 days of daily injection of IL-6 or PBS in C57BL/6 mice (D), from untrained and trained WT and IL-6 KO mice (E) and from WT and IL-6 KO mice after 3 days of cold exposure (F). UCP1 antibody was verified with lysate from retinoblastoma deficient (RB −/−) differentiated to brown adipocytes cells and mouse iWAT (G). UCP1 protein content is normalized to β-actin protein content. Values are mean ± SE, (n = 6–12). #: significantly different from WT (p<0.05).*: significantly different from 22°C (p<0.05). A horizontal line indicates a main effect.

*UCP1:* UCP1 protein content (Fig. 3A) did not change with repeated IL-6 injections compared with PBS.

When examining untrained and trained mice, UCP1 protein content in iWAT was ∼60–70% lower (p<0.05) in IL-6 KO than in WT mice (Fig. 3B).

UCP1 protein content increased (p<0.05) ∼118-fold in WT and ∼9 fold in IL-6 KO mice with 3 days of cold exposure resulting in a genotype difference (p<0.05) between WT and IL-6 KO mice (Fig. 3C).

To verify the UCP1 antibody, lysate from retinoblastoma deficient (RB −/−) cells differentiated to brown adipocytes and iWAT lysate was run on SDS-Page and blotted for UCP1 (Fig 3G).

#### STAT3 phosphorylation

*STAT3:* To investigate IL-6 signaling in iWAT phosphorylation of signal transducer and activator of transcription 3 (STAT3) was measured. Phosphorylation of STAT3 increased (p<0.05) ∼2-fold 1 h after an IP injection of IL-6 (PBS, 0.69±0.06; IL-6, 1.35±0.18) with no difference in the total amount of STAT3 protein (PBS, 0.60±0.07; IL-6, 0.82±0.09). STAT3 phosphorylation normalized to total STAT3 protein content increased (P = 0.195) non-significantly by ∼50%.

### Quadriceps

#### mRNA

**Figure 4:**
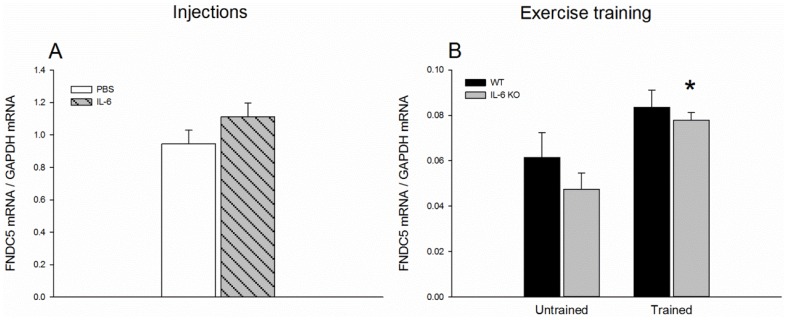
FNDC5 mRNA content in Quadriceps. FNDC5 mRNA content in Quadriceps after 7 days of daily injection of IL-6 or PBS in C57BL/6 mice (A) and in untrained and trained WT and IL-6 KO mice (B). The target mRNA is normalized to GAPDH mRNA content. Values are mean ± SE, (n = 7–12). *: significantly different from untrained (p<0.05).

*FNDC5:* There was no change in FNDC5 mRNA content in quadriceps in response to the repeated IL-6 injections relative to PBS injections (Fig. 4A). Quadriceps FNDC5 mRNA content increased (p<0.05) ∼1.5 fold in the IL-6 KO mice in response to exercise training with no difference in WT mice (Fig. 4B).

## Discussion

The major findings of the present study are that a daily injection of IL-6 for 7 days increased UCP1 mRNA content in iWAT, that IL-6 was required for the exercise training-induced increase in iWAT UCP1 mRNA and that the cold exposure-induced increase in UCP1 protein content observed in WT mice was markedly reduced in IL-6 KO mice. Additionally, when examining untrained and trained mice, there was a lower iWAT UCP1 protein level in IL-6 KO mice than in WT.

The effect of exercise training on iWAT UCP1 mRNA content in WT mice observed in the present study is in agreement with previous studies showing increased iWAT UCP1 mRNA after both swimming and treadmill exercise training [8], [9]. The novel finding that the exercise training-induced increase in iWAT UCP1 mRNA was abolished in IL-6 KO mice adds to the previous observations and indicates that IL-6 plays a role in the regulation of UCP1 mRNA in response to exercise training. The present finding that daily IP injections of IL-6 for 7 days can increase iWAT UCP1 mRNA together with a previous study showing that IL-6 is released from contracting skeletal muscle [10], raises the possibility that IL-6 released from skeletal muscle during exercise signals to increase iWAT UCP1 mRNA content. This is supported by the observed increase in STAT3 phosphorylation in iWAT in response to a single injection of IL-6 with plasma concentrations comparable to that observed in response to exercise [19]. However, the increase in STAT3 phosphorylation may in part be due to an increase in total STAT3 protein content and it cannot be excluded that IL-6 has an indirect effect on iWAT UCP1 mRNA content through other circulating factors such as chemokine (C-X-C motif) ligand 1 (CXCL1) as previously suggested [24].

The observation that UCP1 protein in iWAT did not change in WT in response to exercise training nor in response to repeated injections of IL-6 may indicate that a longer period of exercise training or IL-6 injections is required to increase expression of UCP1 protein. A further consideration is that expression of UCP1 mRNA and protein follow separate time courses [25]. The lower UCP1 protein content in IL-6 KO mice than in WT mice suggests that IL-6 is important for the regulation of UCP1 protein expression in iWAT. Such IL-6 mediated UCP1 regulation may partially explain the decreased body temperature previously observed in IL-6 KO mice when exposed to 4°C for 6 h [17]. Thus it could be suggested that IL-6 is involved in the regulation of cold-induced UCP1 expression in iWAT. This is supported by the present finding that the cold exposure-induced increase in UCP1 protein is markedly reduced in IL-6 KO mice. Additionally UCP1 mRNA content in iBAT (data not shown) was not blunted in the IL-6 KO mice with cold exposure in the present study. Together with a previous study showing similar cold-induced iBAT UCP1 protein content in WT and IL-6 KO mice [17], this suggests that loss of IL-6 mediated UCP1 regulation in iWAT may have contributed to the lower body temperature during cold exposure [17]. However, it must be recognized that the expression of UCP1 in iWAT is much lower than in BAT [25] indicating that iWAT has a much lower thermogenic capacity than BAT. Hence, the decreased iWAT UCP1 protein content in IL-6 KO mice is may be insufficient to explain the lower body temperature previously observed [17], and it could be speculated that the lack of IL-6 has an effect on UCP1 expression in adipose compartments besides iWAT.

The present finding that the plasma concentration of IL-6 did not increase with cold exposure in WT mice is in agreement with previous findings in humans [26]. As the plasma concentration of IL-6 has been shown to increase in response to a single exercise bout [19], this may suggest that IL-6 has different regulatory roles during cold exposure and exercise. This is further supported by the observation that UCP1 mRNA content increased in both WT and IL-6 KO mice in response to cold exposure but only in WT mice in response to exercise training. Because IL-6 mRNA levels has been shown to increase in rat brain with cold exposure [27] the observations that plasma IL-6 does not increase together with the reduced UCP1 protein response in IL-6 KO mice with cold exposure may suggest that IL-6 exerts a central effect during cold exposure. In line with this possibility is the previous finding that overexpression of IL-6 in mouse acinar cells results in resistance to high fat diet-induced increase in bodyweight and body fat [28]. Thus, the present data raise the possibility that IL-6 acts through increasing plasma levels during exercise and centrally or locally during cold exposure to increase expression UCP1 mRNA and protein in iWAT.

The present findings that IL-6 regulates iWAT UCP1 expression could suggest that IL-6 participates in iWAT browning. However, the observation that the mRNA content of Elovl3 and Cidea did not increase in response to IL-6 injections and that there was no difference between WT and IL-6 KO mice provide no evidence that IL-6 is mediating the regulation of Elovl3 and Cidea in iWAT. Additionally, Elovl3 and Cidea mRNA content did not increase significantly in iWAT in response to cold exposure, which is in contrast to previous observations [9] and the lack of change with exercise training does not suggest a role of Elovl3 and Cidea in exercise training induced browning. However, with the low number of samples with detectable Elovl3 and Cidea mRNA it is difficult to draw conclusions.

Based on previous findings that PGC-1α is important in the regulation of UCP1 expression in iWAT in response to exercise training [8], [9], it is possible that IL-6 signals through PGC-1α to exert the regulatory effects on UCP1 expression in iWAT during exercise. Interestingly, in the present study exercise training tended to decrease PGC-1α mRNA content in iWAT. Although this was not significant (p = 0.105), this is supported by the observation that a single exercise bout decreased iWAT PGC-1α mRNA content in mice (Ringholm, Lundgaard and Pilegaard unpublished data). Together with the present findings that iWAT PGC-1α mRNA was increased with cold exposure as previously reported [7], this shows that iWAT PGC-1α mRNA content is regulated differently in response to cold exposure and exercise training and suggests that regulation of UCP1 by IL-6 during exercise is conducted independently of PGC-1α. The additional finding that PGC-1α mRNA content did not increase with repeated injections of IL-6 in the present study further supports a PGC-1α-independent IL-6 effect on UCP1 expression in iWAT. However, previous studies have reported that 24 h of IL-6 incubation can increase PGC-1α mRNA content in adipocyte cell culture [29], [30] and injection of IL-6 can induce PGC-1α in eWAT [31]. Taken together this may suggest that adipose tissue compartment as well as time frame of stimulation may be important for the adipose tissue response to IL-6. It may be considered that the effect of IL-6 could be mediated through increased PGC-1α activity and not through increased PGC-1α transcription as exercise training-induced UCP1 expression has been shown to be dependent on PGC-1α [8]. However, PGC-1α has also been shown to bind to its own promoter to increase transcription [32] and an increased PGC-1α activity would thus be expected to cause an increase in PGC-1α mRNA content, which was not observed in the present study. Additionally, PPARγ works in association with PGC-1α to regulate UCP1 in BAT [7]. Because previously published results have shown that exercise training increased iWAT PPARγ mRNA in IL-6 KO mice while no change was apparent in WT mice [13], PPARγ does not seem to be involved in the observed IL-6 mediated UCP1 expression in iWAT. This supports that the effect of IL-6 on UCP1 regulation is PGC-1α independent and suggests that there may be more than one mechanism capable of inducing UCP1 expression in iWAT in response to exercise training.

It has been suggested that FNDC5 released from skeletal muscle as irisin regulates iWAT UCP1 expression during exercise [9]. Thus, it could be speculated that FNDC5 expression in skeletal muscle was increased by circulating IL-6. However, the present results that FNDC5 mRNA content was similar in WT and IL-6 KO mice, and that IL-6 KO but not WT mice increased FNDC5 mRNA content in skeletal muscle with exercise training show that IL-6 is not required for the basal FNDC5 mRNA content or an exercise training-induced increase in FNDC5 mRNA in mouse skeletal muscle. The observation that repeated injections of IL-6 did not change FNDC5 mRNA content in skeletal muscle further supports this notion. Hence it may be speculated that both myokines contribute to the regulation of UCP1 expression in iWAT in response to exercise training.

Together the present data suggest that IL-6 is important for UCP1 protein expression in iWAT. IL-6 is capable of increasing iWAT UCP1 mRNA content, and IL-6 is required for exercise training-induced increases in iWAT UCP1 mRNA and for cold exposure-induced iWAT UCP1 protein up-regulation. These findings highlight the importance of IL-6 in basal, exercise training and cold exposure-induced iWAT UCP1 expression in iWAT.
